# Association between Exposure to Air Pollution and Total Gray Matter and Total White Matter Volumes in Adults: A Cross-Sectional Study

**DOI:** 10.3390/brainsci10030164

**Published:** 2020-03-13

**Authors:** Lance D. Erickson, Shawn D. Gale, Jacqueline E. Anderson, Bruce L. Brown, Dawson W. Hedges

**Affiliations:** 1 Department of Sociology, Brigham Young University, Provo, UT 84602, USA; lance_erickson@byu.edu; 2 Department of Psychology, Brigham Young University, Provo, UT 84602, USA; bruce_brown@byu.edu (B.L.B.); dawson_hedges@byu.edu (D.W.H.); 3 The Neuroscience Center, Brigham Young University, Provo, UT 84602, USA; jacqueline.kunzelman@gmail.com

**Keywords:** UK Biobank, brain volume, gray matter, white matter, air pollution, PM_2.5_, PM_2.5–10_, PM_10_, nitrogen dioxide, nitrogen oxides

## Abstract

Total brain gray-matter and white-matter volumes can be indicators of overall brain health. Among the factors associated with gray-matter and white-matter volumes is exposure to air pollution. Using data from the UK Biobank, we sought to determine associations between several components of air pollution—PM_2.5_, PM_2.5–10_, PM_10_, nitrogen dioxide, and nitrogen oxides—and total gray-matter and total white-matter volumes in multivariable regression models in a large sample of adults. We found significant inverse associations between PM_2.5_ concentration and total white-matter volume and between PM_2.5_, PM_2.5–10_, PM_10_, nitrogen dioxide, and nitrogen oxide concentrations and total gray-matter volume in models adjusted for age, sex, body-mass index, self-assessment of overall health, frequency of alcohol use, smoking status, educational attainment, and income. These findings of pollutant-associated decreases in total gray-matter and total white-matter volumes are in the context of mean PM_2.5_ concentrations near the upper limit of the World Health Organization’s recommendations. Similarly, mean PM_10_ concentrations were below the recommended upper limit, and nitrogen dioxide concentration was slightly above. Still, there are many areas in the world with much higher concentrations of these pollutants, which could be associated with larger effects. If replicated, these findings suggest that air pollution could be a risk factor for neurodegeneration.

## 1. Introduction

Much of the world’s population is exposed to air pollution [1]. In fact, the World Health Organization estimated that in 2016, 91 percent of people across the world lived where exposure to air pollution exceeded their published standards [2]. In addition to its associations with cardiovascular disease [3,4], emerging research suggests that exposure to air pollution might be associated with changes in brain volume and structure [5]. While the mechanisms by which exposure to air pollution could affect brain volume and structure are not fully understood, a variety of factors suggest possible mechanisms by which exposure to air pollution could affect brain structure and function. Toxins in air pollution could damage the blood-brain barrier, enabling the entry of other toxins into the brain [1], and exposure to air pollution has been associated with increased amyloid beta deposition in the brain [6]. In addition, air pollution has been associated with inflammation and oxidative stress, factors that could also influence brain structure and function [7]. Exposure to air pollution has been associated with altered cortical development [8] and brain structure [5]. In addition to possible associations between exposure to air pollution and reduced volume in specific brain regions, such as smaller hippocampal volume [9], exposure to air pollution might also be associated with reduced overall gray-matter and white-matter volume. In this regard, exposure to air pollution has been associated with smaller white-matter volume in children and adult women [10,11,12], with reduced gray-matter and white-matter volume in older adults [11], and with reduced total cerebral volume in older adults [13]. 

Total gray-matter and white-matter volumes appear related to cognitive function and cognitive decline. Although the effect sizes tend to be small, brain volume has also been linked directly to cognitive functioning [14], and larger brain volumes are related to better cognitive functioning in both children and older adults [15,16], whereas smaller brain volumes have been associated with cognitive decline in older women [17] and with dementia incidence in a population-based study [18]. Accordingly, factors such as air pollution that might affect total gray-matter and white-matter volumes could be important in determining individual and public cognitive health. 

Widespread exposure to air pollution in many regions of the world [7], biologically plausible mechanisms by which air pollution could affect brain structure, emerging findings showing associations between exposure to air pollution and smaller gray-matter and white-matter volumes, and the potential role of brain volume in cognitive health indicate the need for additional characterization of the associations between exposure to air pollution and total gray-matter and white-matter volumes, while accounting for other variables associated with brain volume. To further examine the relationship between air pollution and total gray-matter and total white-matter volumes, and to explore the association between exposure to air pollution and brain volume in middle-aged and late middle-aged adults exposed to modest levels of air pollution, we examined the associations between an array of potential toxins found in air pollution—particulate matter <2.5 µm (PM_2.5_), particulate matter 2.5–10 µm (PM_2.5–10_), particulate matter <10 µm (PM_10_), nitrogen dioxide (NO_2_), and nitrogen oxides (NO_x_)—and total white-matter volume and total gray-matter volume using data from the UK Biobank Resource, a large, community-based sample of middle-aged and late-middle-aged women and men in the United Kingdom that includes a range of available variables to allow adjusted statistical models. Consistent with the findings of earlier research, we hypothesized that air pollution would be negatively associated with total gray-matter volume and with total white-matter volume. The UK Biobank sample is somewhat younger than the samples in many of the previous studies examining associations between exposure to air pollution and gray-matter and white-matter volumes, providing information about these associations across a wider age range. Because of previous findings showing associations between air pollution and gray-matter and white-matter volumes [10,11], we estimated separate models for gray-matter and white-matter volumes. 

## 2. Materials and Methods

### 2.1. Study Sample

For our study sample, we used the UK Biobank, a dataset of approximately 500,000 UK adults sampled via population-based registries (http://www.ukbiobank.ac.uk). The UK Biobank received ethical approval from the National Research Ethics Service Committee North West–Haydock (reference 11/NW/0382). All participants provided informed consent and were aged from approximately 40 to 69 years of age at the time of enrollment (http://biobank.ctsu.ox.ac.uk/crystal/field.cgi?id=200). Participants were recruited from across the United Kingdom and initial enrollment was carried out from 2006 to 2010. Participants provided sociodemographic, cognitive, and medical data via questionnaires and physical assessments. Starting in 2014, a subset of the original sample later underwent magnetic-resonance brain imaging (MRI) (UK Biobank Brain Imaging Documentation, http://www.ukbiobank.ac.uk). The MRI data used in the current study were acquired between 2014 and 2019. There were 21,407 participants with processed MRI data at the time that we received regulatory approval from the UK Biobank, and these data were made available to us (UK Biobank Resource under Application Number 41,535). The analyses here include all participants with processed MRI data including total gray-matter and white-matter volumes who had no missing data concerning exposure to air pollution and the preidentified covariates (see below). Our final sample included 18,292 participants with an average age of 62 (range of 44–80). Table 1 shows additional demographics of the sample. 

### 2.2. Measures

#### 2.2.1. Brain Volume 

The UK Biobank used a standard Siemens Skyra 32-channel 3T scanner (Siemens Medical Solutions, Germany) for all magnetic-resonance brain imaging, with 1 × 1 × 1 resolution and a view field of 208 × 256 × 256. For our analyses, we used numerical volume data from the UK Biobank from preprocessed three-dimensional magnetization for rapid echo-gradient (3D MP-RAGE) T1-weighted image derived phenotypes. Full imaging information for the UK Biobank magnetic resonance imaging is available from UK Biobank Brain Imaging Documentation [19,20] (http://biobank.ctsu.ox.ac.uk/crystal/label.cgi?id=100). We used structural numerical volume estimates from image-derived phenotypes from preprocessed total gray matter and total white matter normalized for head size from the UK Biobank image processing pipeline. 

#### 2.2.2. Air Pollution

The UK Biobank incorporated estimates of air-pollution exposure from the Small Area Health Statistics Unit (http://www.sahsu.org), which is associated with the BioShaRE-EU Environmental Determinants of Health Project (https://biobank.ndph.ox.ac.uk/showcase/label.cgi?id=115). The estimates we used represent address-level, mean concentrations of pollutants in 2010, derived from a land-use regression model from the European Study of Cohorts for Air Pollution Effects (http://www.escapeproject.eu/) and traffic data for 2008 using land-use regression modeling from Eurostreets (http://biobank.ctsu.ox.ac.uk/crystal/label.cgi?id=114). Additional information about the estimation of exposure to particulate matter and NO_s_ and NO_x_ is available elsewhere [21,22]. In our analyses, we used estimates of PM_2.5_, PM_2.5–10_, and PM_10_ and estimated concentrations of NO_2_ and NO_x_ (Table 1). 

#### 2.2.3. Covariates

To adjust statistical models for potential confounding, we identified variables that could be associated with gray-matter and white-matter volumes based on their associations with cognitive function or possible associations with brain volume. Accordingly, we included age, sex, body-mass index, a respondent self-assessment of overall health, frequency of alcohol use, smoking status, educational attainment, and income [23,24,25,26] as covariates in the statistical models. 

### 2.3. Statistical Analyses

The independent variables of interest were PM_2.5_, PM_2,5–10_, PM_10_, NO_2_ and NO_x_; the dependent variables were total gray-matter volume and total white-matter volume, with separate models for total gray matter and for total white matter. Because of potential collinearity between the measures of air pollution, we estimated separate Ordinary Least Squares multivariable regression models for each measure of air pollution, each adjusting for the predetermined potentially confounding variables. We also estimated a series of interaction models for total gray and white matter with each measure of air pollution, that included an interaction of air pollution with age, educational status, sex, and overall health, respectively. For all analyses, we used Stata 15.2 (StataCorp, Stata Statistical Software, Release 15. College Station, TX, USA). 

## 3. Results

Women comprised 52 percent of the sample and men 48 percent. The mean age was 62.15 years (standard deviation: 7.44; range: 44 to 80 years). The mean total gray-matter volume was 796,316 mm^3^ (standard deviation: 47,940), and the mean total white-matter volume was 708,111 mm^3^ (standard deviation: 40,696). The mean PM_2.5_ concentration was 9.90 μg/m^3^ (standard deviation: 1.01; range: 8.17 to 19.65) (Table 1). 

In the adjusted models (Table 2), total gray-matter volume was inversely associated with PM_2.5_ (b = −659, *p <* 0.05), PM_2.5–10_ (b = −633, *p <* 0.05), PM_10_ (b = −528, *p <* 0.001), NO_2_ (b = −103, *p <* 0.01), and NO_x_ (b = −42, *p <* 0.05). Total white matter was also inversely associated with PM_2.5_ (b = −579, *p <* 0.05) but not with other pollutants (Table 2). There were no interactions between the air pollutants and age concerning total gray-matter or total white-matter volumes (Table 3). There was only one interaction between air pollution and sex concerning gray-matter volume, NO_2_ (b = 161, *p <* 0.05), and none concerning white matter (Table 4). There were no interactions between the air pollutants and education concerning total gray-matter or total white-matter volumes (Table 5). Finally, there was one interaction between pollution and the overall health rating concerning white-matter volume PM_2.5–10_ (b = 1044, *p <* 0.05) (Table 6).

## 4. Discussion

The primary findings from this study of 18,292 participants with a mean age of 62.15 years from the UK Biobank were the associations between PM_2.5,_ PM_2.5–10,_ PM_10,_ NO_2,_ and NO_x_ concentrations and total gray-matter volume and the association between PM_2.5_ and total white-matter volume, in models adjusted for age, ethnic background, sex, educational attainment, household income, an estimate of self-rated overall health, body-mass index, frequency of alcohol use, and smoking status. Although these effect sizes are small, they do suggest that these pollutants might contribute to loss of gray-matter volume and white-matter volume in community-dwelling adults. Because these effects occurred in models adjusted for age, they also suggest that this volume loss is independent of any age-related loss of brain volume. To place the size of these findings in context, prior work [27] has shown an approximately 0.5% per year decrease in brain volume related to aging starting around the age of 60 (the mean age in this study was 62). In our models, we found that for every one-unit increase in PM_2.5_, total gray-matter volume decreased by 659 mm^3^ (Table 2), which is a 0.08% decrease for every one-unit increase in PM_2.5_, or a 0.40% decrease for every five units. That is, a five-unit increase in PM_2.5_ is approximately 80% of the yearly effect of aging on the brain in this age range.

These findings showing associations between air pollution and total gray-matter and total white-matter volumes are consistent with previous studies that found associations between exposure to air pollution and brain changes [1,5,11]. Specifically, a study of older women from the Women’s Health Initiative Memory Study (WHIMS; age range 71 to 89 years; *N* = 1403) found that PM_2.5_ was associated with reduced white-matter volume and total brain volume but not gray-matter volume [10]. However, in addition to the fact that this study focused on a different age range from our study and only included women, the authors separated participants in the WHIMS into quartiles of cumulative air pollution averages, concentrations that were somewhat higher than the concentrations in our study (highest quartile of PM_2.5_ was 14 to 22 μg/m^3^ and lowest quartile, 6 to11 μg/m^3^ compared to the PM_2.5_ mean concentration of 9.90 μg/m^3^ in our study). Another study of older adult women (average age = 70; *N* = 1365), also using participants collected from the WHIMS, found regions of decreased white-matter and gray-matter volume associated with PM_2.5_ [28]. Similarly, in a study of older female and male adults (median age = 68 years; *N* = 943) from the Framingham Offspring Study, there was an association between higher PM_2.5_ (median level 11.1 μg/m^3^) and lower total cerebral brain volume [13]. This study did not investigate total white-matter volume or total gray-matter volume. Finally, a study using participants from the Atherosclerosis Risk in Communities (ARIC) study (average age =76 years; *N* = 1753), which consisted of cohorts in four different US states, found lower volumes of subcortical gray matter structures were associated with higher PM_2.5_ across groups but in no other brain volume measures. However, the study found an association between PM_2.5_ and smaller regional brain volumes in one of the four sites [29]. While there are differences between these studies and ours in terms of age of the participants, air pollution exposure (including which pollutants were measured), sample size, covariates, country of origin (ours is from the United Kingdom, whereas the other studies had samples from the United States) and even volumetric methods, our findings are broadly consistent with these studies in that all show an association between exposure to air pollution and smaller brain volume. Further, our findings are from a sample slightly younger than the ones in the WHIMS and ARIC studies. Finally, our findings showing associations between exposure to air pollution and smaller gray-matter and white-matter volumes are also consistent with previous studies reporting associations between exposure to air pollution and smaller brain volume in children [30]. 

The associations we found between several components of air pollution and brain volume were in a sample from the United Kingdom, where the mean PM_2.5_ concentration of 9.90 μg/m^3^ was near the upper acceptable limit recommended by the World Health Organization of 10 μg/m^3^ [2]. Other regions of the world can have far higher PM_2.5_ concentrations [7]. In the UK Biobank sample, the mean PM_10_ concentration was below the recommended upper limit, and the nitrogen-dioxide concentration was below the upper acceptable limit [2]. Our findings, therefore, suggest that even comparatively low levels of exposure—concentrations slightly below and above the World Health Organization’s recommended upper limit—to air pollution could be associated with reduced total gray-matter and total white-matter volumes, which (even though we found small amounts of volume loss) is concerning given the widespread distribution of air pollution [1]. However, the cognitive and neuropsychiatric significance of the amount of volume loss we found associated with exposure to air pollution at the levels used in our study is unknown. In a study also based on data from the UK Biobank, there was only a small association between exposure to air pollution and cognitive function [31], although the association between the amounts of gray-matter and white-matter volume loss that we found and cognitive function, dementia risk, and neuropsychiatric function requires additional research, including in regions where concentrations of air pollutants are higher than in the UK Biobank sample. 

In our interaction modes, we found only two significant interactions—the interaction between self-estimated overall health and PM_2.5–10_ predicting total white-matter volume, and the interaction between sex and NO_2_ concentration predicting total gray-matter volume. While these significant interactions could be due to chance, in that we ran multiple interaction models, they do provide some evidence that overall health and sex could moderate the effects of air pollution on white-matter and gray-matter volumes, although these possible associations require additional study. There were no interactions between any of the pollutants and age or educational attainment in predicting total gray-matter or total white-matter volumes. 

Although we did not design our study to identify mechanisms underlying the associations we found between air pollution and total gray matter and white matter volumes, several biologically plausible mechanisms could account for these associations. Toxins in air pollution can damage the blood-brain barrier [1], possibly enabling toxin entry into the brain, some of which could also enter the brain directly through the olfactory nerve. Additionally, brain amyloid might be higher in people who live in areas with high exposure to air pollution compared to people with lower levels of exposure [6]. Air pollution is also associated with inflammation and oxidative stress, factors that can adversely affect the brain [7]. Finally, air pollution such as particulate matter has also been associated with alterations in gene expression [32]. Thus, air pollution has been associated with multiple pathways that may adversely affect the central nervous system [33].

While this study has several strengths, such as a large, community-based sample, objective independent and dependent variables, and adjustment for multiple potentially confounding variables, several limitations require consideration. Because we used air-pollution data from just one year, there is the potential for misclassification of exposure data in that some participants may have previously lived in areas with different levels of exposure to air pollution than where they lived when the exposure was measured. This could be particularly problematic in that outcomes such as total gray-matter and total white-matter volumes might reflect cumulative lifetime exposures. Related to this problem is our use of estimates of air pollution at residential addresses without considering the time participants spent away from home. We also did not include indoor-air pollution in our models, which could also affect exposure history. In short, our exposure variable likely captured only part of the total possible exposure to air pollution. There was also a gap between the estimate of the exposure to air pollution (2010) and the brain imaging (2014 to 2019), which could have affected the associations we found between air pollution and total white-matter and gray-matter volumes. An additional potential limitation is that not all participants in the UK Biobank study had available brain imaging data, resulting in the possibility of selection bias. The study’s cross-sectional design precludes a determination of cause and effect, and we might not have included all the relevant covariates into our models, resulting in the possibility of residual confounding. 

In conclusion, in this large sample from the UK Biobank with a mean age of 62.15 years, we found inverse associations between PM_2.5,_ PM_2.5–10,_ PM_10,_ NO_2,_ and NO_x_ concentrations and total gray-matter volume, and between PM_2.5_ concentration and total white-matter volume in adjusted models. In the context of the limitations associated with this study, the findings suggest that exposure to components of air pollution might be associated with decreases in total gray-matter and white-matter volumes at mean PM_2.5_ concentrations near the upper limit of the World Health Organization’s recommendations. If replicated, these findings suggest that air pollution could be a risk factor for neurodegeneration. Additional research is needed to determine the clinical ramifications of this on cognition, and the risk of dementia associated with the amounts of total gray-matter and total white-matter volume loss we found. 

## Figures and Tables

**Table 1:** Descriptive statistics of study variables.

	Mean or Proportion ^a^	Standard Deviation	Minimum	Maximum
Total brain volume (mm^3^)				
Gray matter	796,316.27	47,940.61	548,842.00	956,930.00
White matter	708,111.03	40,696.72	568,495.00	915,171.00
Air pollution µg/m^3^)				
Particulate matter_2.5_	9.90	1.01	8.17	19.65
Particulate matter_2.5__–__10_	6.36	0.88	5.57	10.25
Particulate matter_10_	16.01	1.85	11.78	25.21
Nitrogen dioxide	25.61	6.86	12.93	89.55
Nitrogen oxides	42.33	14.02	19.74	231.89
Age (years)	62.15	7.44	44.00	80.00
Female	0.52		0.00	1.00
Race				
White	0.97		0.00	1.00
Black	0.01		0.00	1.00
Asian	0.01		0.00	1.00
Other	0.01		0.00	1.00
College degree	0.50		0.00	1.00
Household income (in pounds)				
<18,000 k	0.12		0.00	1.00
18,000 k–30,999 k	0.28		0.00	1.00
31,000 k–51,999 k	0.31		0.00	1.00
52,000 k–100,000 k	0.23		0.00	1.00
>100,000 k	0.06		0.00	1.00
Overall health	2.99	0.66	1.00	4.00
Body-mass index (weight in kg/height in meters squared)	26.59	4.42	13.39	58.70
Smoking status				
Non-smoker	0.63		0.00	1.00
Past	0.33		0.00	1.00
Current	0.04		0.00	1.00
Drinking frequency				
Daily or almost daily	0.17		0.00	1.00
3–4 times/week	0.28		0.00	1.00
Once or twice/week	0.27		0.00	1.00
1–3 times/month	0.12		0.00	1.00
Special occasions	0.10		0.00	1.00
Never	0.06		0.00	1.00

Note: ^a^ The number in the Mean or Proportion column is a proportion when there is nothing listed in the Standard Deviation column. *N* = 18,292. Source: UK Biobank.

**Table 2:** Associations between total brain gray-matter and white-matter volumes (mm^3^) and air pollution: unstandardized coefficients and 95% confidence intervals from linear regression.

	Gray Matter		White Matter	
	b	95%CI	b	95%CI
PM_2.5_				
Unadjusted	838 *	151, 1524	243	−340, 826
Adjusted	−659 *	−1179, −140	−579 *	−1144, −14
PM_2.5 to 10_				
Unadjusted	−549	−1334, 237	410	−257, 1077
Adjusted	−633 *	−1218, −47	142	−495, 779
PM_10_				
Unadjusted	−307	−682, 68	189	−129, 508
Adjusted	−528 ***	−809, −248	−22	−328, 283
Nitrogen Dioxide				
Unadjusted	195 ***	94, 296	83	−3, 169
Adjusted	−103 **	−180, −26	−72	−155, 12
Nitrogen Oxides				
Unadjusted	88 ***	39, 138	39	−3, 81
Adjusted	−42 *	−79, −4	−26	−67, 15

Note: adjusted models include age, sex, race, education, income, overall health, BMI, smoking status and frequency of drinking alcohol. N = 18,292. Source: *UK Biobank*. * *p* < 0.05, ** *p* < 0.01, *** *p* < 0.001.

**Table 3:** Interactions between pollution (µg/m^3^) and age concerning total gray-matter and white-matter volumes (mm^3^): unstandardized coefficients and 95% confidence intervals from linear regression.

	Gray Matter		White Matter	
	b	95%CI	b	95%CI
PM_2.5_				
Pollutant	−1771	−6115, 2572	−519	−5243, 4204
Age	−3723 ***	−4416, −3030	−1572 ***	−2325, −819
Interaction	18	−52, 87	−1	−76, 75
PM_2.5 to 10_				
Pollutant	−1263	−6162, 3636	109	−5219, 5436
Age	−3602 ***	−4105, −3098	−1576 ***	−2124, −1029
Interaction	10	−68, 88	1	−85, 86
PM_10_				
Pollutant	−2441 *	−4808, −74	−277	−2853, 2298
Age	−4035 ***	−4645, −3425	−1639 ***	−2303, −975
Interaction	31	−7, 68	4	−37, 45
Nitrogen Dioxide				
Pollutant	−305	−946, 335	−105	−801, 592
Age	−3631 ***	−3906, −3357	−1595 ***	−1894, −1296
Interaction	3	−7, 14	1	−11, 12
Nitrogen Oxides				
Pollutant	−212	−527, 103	−103	−445, 239
Age	−3662 ***	−3889, −3434	−1632 ***	−1879, −1384
Interaction	3	−2, 8	1	−4, 7

Note: models include age, sex, race, education, income, overall health, BMI, smoking status and frequency of drinking alcohol. *N* = 18,292. Source: *UK Biobank*. * *p* < 0.05, *** *p* < 0.001.

**Table 4:** Interactions between pollution (µg/m^3^) and sex concerning total brain volume (mm^3^): unstandardized coefficients and 95% confidence intervals from linear regression.

	Gray Matter		White Matter	
	b	95%CI	b	95%CI
PM_2.5_				
Pollutant	−1113 **	−1858, −369	−796	−1606, 14
Female	17,612 ***	7429, 27,796	−14,070 *	−25,145, −2995
Interaction	870	−152, 1893	417	−695, 1529
PM_2.5 to 10_				
Pollutant	−942 *	−1778, −107	222	−687, 1130
Female	22386 ***	14,884, 29,888	−8921 *	−17,079, −762
Interaction	607	−561, 1775	−155	−1426, 1115
PM_10_				
Pollutant	−700 ***	−1097, −302	−26	−458, 407
Female	20,793 ***	11,800, 29,787	−10,022 *	−19,806, −238
Interaction	339	−219, 897	7	−600, 613
Nitrogen Dioxide				
Pollutant	−186 ***	−295, −76	−108	−227, 11
Female	22,108 ***	18,097, 26,119	−11,739 ***	−16,102, −7377
Interaction	161 *	10, 312	70	−94, 234
Nitrogen Oxides				
Pollutant	−77 **	−131, −24	−38	−96, 21
Female	23,325 ***	20,019, 26,631	−10,861 ***	−14,457, −7265
Interaction	69	−5, 143	22	−58, 102

Note: models include age, sex, race, education, income, overall health, BMI, smoking status and frequency of drinking alcohol. *N* = 18,292. Source: *UK Biobank*. * *p* < 0.05. ** *p* < 0.01. *** *p* < 0.001.

**Table 5:** Interactions between pollution (µg/m^3^) and educational attainment on total brain volume (mm^3^): unstandardized coefficients and 95% confidence intervals from linear regression.

	Gray Matter		White Matter	
	b	95%CI	b	95%CI
PM_2.5_				
Pollutant	−543	−1293, 207	−602	−1418, 214
Education	−1410	−11,592, 8772	−2121	−13,194, 8952
Interaction	−220	−1243, 803	44	−1068, 1157
PM_2.5 to 10_				
Pollutant	−599	−1392, 193	130	−732, 992
Education	−3203	−10,743, 4337	−1880	−10,080, 6321
Interaction	−73	−1247, 1101	27	−1250, 1304
PM_10_				
Pollutant	−444 *	−834, −53	−51	−476, 374
Education	−875	−9882, 8132	−2672	−12,470, 7126
Interaction	−175	−733, 384	60	−548, 667
Nitrogen Dioxide				
Pollutant	−50	−163, 62	−46	−168, 77
Education	−1104	−5113, 2904	−462	−4821, 3898
Interaction	−96	−248, 55	−48	−212, 117
Nitrogen Oxides				
Pollutant	−34	−87, 20	−33	−91, 25
Education	−2945	−6241, 351	−2260	−5844, 1324
Interaction	−15	−89, 59	13	−67, 94

Note: models include age, sex, race, education, income, overall health, BMI, smoking status and frequency of drinking alcohol. *N* = 18,292. Source: *UK Biobank*. * *p* < 0.05.

**Table 6:** Interactions between pollution (µg/m^3^) and self-rated overall health concerning total brain volume (mm^3^): unstandardized coefficients and 95% confidence intervals from linear Regression.

	Gray Matter		White Matter	
	b	95%CI	b	95%CI
PM_2.5_				
Pollutant	−642	−2986, 1701	−68	−2616, 2480
Overall Health	1839	−5806, 9484	2114	−6200, 10,428
Interaction	−6	−770, 759	−171	−1002, 660
PM_2.5 to 10_				
Pollutant	1162	−1538, 3862	−2984 *	−5920, −48
Overall Health	5626	−48, 11,300	−6230 *	−12,400, −60
Interaction	−600	−1480, 281	1044 *	87, 2002
PM_10_				
Pollutant	198	−1090, 1486	−1242	−2643, 160
Overall Health	5688	−1103, 12,479	−6109	−13,496, 1278
Interaction	−243	−663, 177	408	−50, 865
Nitrogen Dioxide				
Pollutant	−159	−502, 184	−90	−463, 283
Overall Health	1289	−1742, 4321	255	−3042, 3552
Interaction	19	−93, 131	6	−116, 128
Nitrogen Oxides				
Pollutant	9	−155, 173	−49	−227, 129
Overall Health	2517 *	43, 4991	87	−2603, 2777
Interaction	−17	−71, 37	8	−51, 66

Note: models include age, sex, race, education, income, overall health, BMI, smoking status and frequency of drinking alcohol. *N* = 18,292. Source: *UK Biobank*. * *p* < 0.05.
